# Traversing arbuscular mycorrhizal fungi and *Pseudomonas fluorescens* for carrot production under salinity

**DOI:** 10.1016/j.sjbs.2021.06.025

**Published:** 2021-06-24

**Authors:** Vinod Kumar Yadav, Radha Krishna Jha, Prashant Kaushik, Fahad H. Altalayan, Thamer Al Balawi, Pravej Alam

**Affiliations:** a University Department of Botany, Ranchi University, Ranchi 834001, Jharkhand, India; b Instituto de Conservación y Mejora de la Agrodiversidad Valenciana, Universitat Politècnica de València, Camino de Vera 14, Valencia 46022, Spain; c Department of Biology, College of Science and Humanities, Prince Sattam bin Abdulaziz University, Al-Kharj 11942, Saudi Arabia

**Keywords:** Carrot, *Gigaspora gigantea*, Microbes, Mycorrhizal fungi, *Pseudomonas fluorescens*, Soil

## Abstract

Carrot is a vital supply of dietary fiber, vitamins, and carotenoids, and it is also rich in antioxidants and minerals. Soil salinity significantly reduces the yield and quality of carrots. Mycorrhiza inoculum (AMF) is known to improve morphological and biochemical traits of vegetables even under saline conditions. But the role of AMF in combating soil salinity effect in carrot is not studied in detail. Therefore here, in the first set, carrot seeds are inoculated with microbes in a pot experiment under polyhouse condition. In total, we applied 7 treatments with different combinations of Mycorrhiza inoculum (*Glomus mosseae* (G_m_) and *Gigaspora gigantea* (G_g_)) and phosphate solubilizing bacteria (*Pseudomonas fluroscens* (P_f_)). In pot experiment study the best two treatments were the combination of G_m_ + P_f_ + G_G_ and P_f_ + G_G_. Both of these treatments were selected for validation under the open field conditions. Primarily, there seems to be a promising opportunity for AMF application to carrots under pot culture as well as under field trials because of promising effect towards morphological parameters, especially root weight, and disparities in nutrients and metabolites. Overall, our study highlights mycorrhizal fungi and other microbes' efficacy in achieving a successful carrot production under salinity stress.

## 1 Introduction

Carrot (*Daucus carota* L.) is a native of Western Asia (Afghanistan), and botanically carrot is more a biennial herb which is extensively cultivated as an annual vegetable. Carrot varieties are classified as European/Temperate types and Asiatic/tropical types based on their temperature requirements at the time for bolting. Carrots generally grown are orange colored, but white and red colors are also cultivated based on local preferences (Simon et al., 2019). The leaf is compound, highly dissected but earlier it is unbranched. The carrot storage root is an excellent supply of dietary fiber, vitamins, and carotenoids, and it is also a rich source of antioxidants and minerals (Stolarczyk and Janick, 2011). With rising consumer awareness for bioactive foods, carrots are becoming very popular because of their plentiful benefits and nutritional value to human health.

Carrots researchers are focused on growth, nutrient content, tissue culture, breeding, and then carotenoid synthesis regulation in the cultivated carrots. But abiotic stress such as salinity is one of the most substantial challenges and a growing problem for agriculture worldwide (Shrivastava and Kumar, 2015, Ahmad et al., 2019, Ahanger et al., 2020). Salinity stress also disturbs the osmotic and nutrient balance, stomatal and hydraulic conductance; therefore, net photosynthetic efficiency (Füzy et al., 2019).

Mycorrhiza inoculum is known for improving morphological and biochemical traits in vegetables, including carrots (Regvar et al., 2003). Plants are usually colonized both by arbuscular mycorrhizal (AMF) fungi and with mycorrhization, both are benefitted (Kiers and West, 2015). Moreover, in some cases, greater tolerance to biotic and abiotic stresses has been confirmed as the result of mycorrhizal inoculum (Malhi et al., 2021). AMF are identified to have a substantial impact on host-plant physiology and biochemistry, along with secondary metabolites (Kaushik et al., 2020). Bacteria, particularly phosphate solubilizing bacteria (PSB), *Pseudomonas fluorescens*, is an essential bacterium in the rhizosphere for optimum plant growth and development and also works in coordination with the AMF. Therefore, AMF and PSB combination might ameliorate plant development and maybe a chemical fertilizer ecofriendly alternate (Massa et al., 2020, Sbrana et al., 2014). This symbioses can improve plant performance under several biotic and abiotic stresses.

Under salt stress, plants suffer from Na^+^ toxicity (Ahmad et al., 2017, Porcel et al., 2016). Over-accumulation of Na^+,^ which corresponds to deficiency of K^+^ in crops results in the damages of cellular organelles, disrupts osmotic potentials, and impairs photosynthetic efficiency (Ahmad et al., 2010, Pozo et al., 2015). In this respect, biological inoculations which can be eco-friendly such as mycorrhizal applications can be an alternative to alleviate salt stress. AM symbiosis helps the plant by favoring uptake of nutrients and water and induces noteworthy changes in host traits, such as increased root architecture, restoration of degraded land, and especially in stress resistance (Liu et al., 2016). AMF colonization improved biomass in *Gossypium hirsutum*, *Elaeagnus angustifolia* and *Chrysanthemum morifolium* (Liu et al., 2016, Chang et al., 2018, Wang et al., 2018). It has also been described that AMF and PSB can trigger nonenzymatic defense systems and phytohormones synthesis during salt stress (Ahmad et al., 2015a, Ahmad et al., 2015b, Navarro et al., 2013). Porcel et al., 2016 established a relationship between OsSOS_1_, OsHKT_2_, OsNHX_3_, etc. and salt stress, when roots were subjected to mycorrhization. Additionally, these below-ground microbes modify the unfavorable environment to stimulate mineral cyclization and mineralized soil nutrients; especially P, N and K content (Jacoby et al., 2017).

The very significant point to select useful soil microbes is that they are not host-specific and can be readily (Sharma et al., 2018). However, many efforts have been made to prove the utility of microbes in hydroponic greenhouses. AMF and PSB combination is now emerging and gaining popularity as they not only accelerate mineral nutrition but also repel any pathogenic attack as well as increase tolerance to abiotic stresses (Verbon and Liberman, 2016). Together this information can provide an elite microbial inoculum that can lift the plant nutrition that could favour crops-soil interface (Xia et al., 2015). Therefore, the present study was carried out with a popular carrot variety to determine AMF and PSB's effect on the morpho-biochemical attributes of the carrot cultivated under saline conditions.

## 2 Material and methods

### 2.1 Experimental design

The experiment was conducted over a period of two years (2018–2019). In the first year/season (October 2018), a pot experiment was performed in order to evaluate the best treatment for carrot growth in a pot experiment under polyhouse condition. After 80–90 days of sowing, plants in the pot experiment were subjected to various morpho-physiological characterizations. Whereas some plants were not harvested and left out for seed formation in the next season (during February). Later, seeds were collected and stored in fumigated glass jars that were sown in the next cropping season (October 2019), in natural field conditions. The best two treatments from the pot experiment were selected for testing under open field conditions.

The pot experiment was conducted inside a polyhouse (26 ± 4 °C and 56–60% relative humidity) situated at the Department of Botany, Ranchi University coordinated at 23° 22′ 18″ N, 85° 19′ 27″ E, Ranchi Jharkhand, India. The variety used was ‘Hisar Gairic’ and seeds were sown in the first week of October 2018 in a randomized complete block design in 3 replications with 15 plants in each replication. A mixture of soil and sand in 3:1 was used for cultivation, constituting of 71.8% sand, 23.5% silt, and 4.0% clay. The soil's chemical composition was 0.051% N, 0.026% P, 0.058% organic carbon and a pH of 6.4 (Bandyopadhyay et al., 2012). Furthermore, the soil sand mixture was filtered through a 2-mm sieve and sterilized using autoclave at 121 °C to eradicate the previous microbial strains present in the soil.

### 2.2 Microbial inoculum and salt treatment

*Glomus mosseae* inoculum with 80–86 percent colonization (root parts) and 780–800 AM spores (w/w) was obtained from the Department of Botany, Kurukshetra University Kurukshetra, and *Gigaspora gigantea* inoculum with 75–79 percent colonization (root parts) and 870–890 AM spores (w/w) was obtained from the Forest Pathology Discipline, FRI, Dehradun. Each inoculum was introduced at a rate of 100 g per pot, for a total of 50 + 50 g (*G. mosseae* + *G. gigantea*) for dual and consortium treatments (Saini et al., 2020a, Saini et al., 2020b). *Pseudomonas fluorescens* (ATCC-17400) was obtained from the CSIR-Institute of Microbial Technology (CSIR-IMtech) in Chandigarh, India. For its application, the roots were soaked in the nutrient broth medium for 10 min (Saini et al., 2020a, Saini et al., 2020b). The second application for *P. fluorescens* was also provided by pouring broth media comprising a bacterial colony over the roots. Table 1 demonstrates the treatments studied along with their codes. One plot was left for control where no inoculation and no salinity stress were given and similarly one plot was having only those plants which had salt stress. The other two plots have salt and microbial inoculation, as shown above in Table 2. Salt stress was supplied to plants based on the method described earlier by Bano et al. (2012). Briefly, 150 mM NaCl inhibits the plants growth compared to 50 and 100 mM NaCl. So, based on this observation, salt stress of 15 mM NaCl was given after 10 days of germination and thereafter supplied at weekly intervals with Hoagland’s nutrient solution (Velikova et al., 2000).

**Table 1:** Treatment and inoculums of pot polyhouse experiment.

**Treatment**	**Microbial inoculation**
C	Control
T1	*Glomus mosseae* (G_m_)
T2	*Pseudomonas fluorescens* (P_f_)
T3	*Gigaspora gigantea* (GG)
T4	G_m_ + P_f_
T5	G_m_ + GG
T6	P_f_ + GG
T7	G_m_ + P_f_ + G_G_ (consortium)

**Table 2:** Treatment and inoculums of under open field conditions.

**Treatment**	**Microbial inoculation**
C	Control (No inoculum; no salt)
S	Control (Salt concentration only)
SPG	Salt + P_f_ + G_G_†
SGPG	Salt + G_m_ + P_f_ + G_G_

†G_m_ – Glomus mosseae, P_f_ – *Pseudomonas fluorescens*, G_G_ – *Gigaspora gigantea*.

### 2.3 Plant characterization and data analysis

Plant characterization was performed after 80 days of sowing. Root and shoot length of all the plants in replication were measured at maturity. The total chlorophyll and carotenoid content were estimated to Arnon’s method (Arnon, 1949). Arbuscular mycorrhizal fungi (AMF) and AM root colonization (%) were determined based on the methods defined elsewhere (Phillips and Hayman, 1970, Giovannetti and Mosse, 1980). Some pots were intentionally remained undisturbed so that they develop seeds, and that were collected for the next round of experimentation under open field conditions.

### 2.4 Field experiment layout

Our second level of the experiment was performed under open field conditions, where seeds were collected in February 2019 from previous experimented carrot pot plants and sown in early October 2019. At first, a field of 2.5 × 2.5 m was ploughed thoroughly and around 3–5 cm layer of sanitized soil: sand (3:1) mixture was evenly distributed, and the field is divided into four 1 × 1 m plots/plant-beds with 15 cm alleyways. Furrows of 15–20 cm were made, and carrot seeds were sown. Each plant-bed had 2 furrows with five plants each. A proper drip irrigation system was installed so that each plot receives an equal amount of watering.

### 2.5 Plant characterization under open field

Ten random plants were selected for morphological and biochemical analysis after 90 days for sowing. For peroxide (H_2_O_2_) content (Velikova et al., 2000) method was used, a 0.5 g of leaf sample was ground and treated with 5 ml of trichloroacetic acid (0.1%, w/v). It was then centrifuged at 12,000*g* for 15 min, 0.5 ml of supernatant was mixed with an equal amount of 10 mM potassium phosphate buffer, and 1 ml of 1 M potassium iodide (KI) and absorbance was taken at 390 nm. Electrolyte leakage or inorganic ion in leaves was estimated by the Dionisio-Sese and Tobita method (Dionisio-Sese and Tobita, 1998).

Proline content was determined by the method of Bates et al. (1973). For enzymatic activity determination 10 g of fresh leaves samples were homogenized with100 mM Tris-HCl, 1 mM EDTA, 10 mM MgCl_2_, 5 mM magnesium acetate, 5 mM DTT, and 1.5% PVP-40. The mixture was sieved with muslin cloth and then centrifuged at 10,000*g* for 15 min. The supernatant was taken for further enzyme assay, supplemented with serine, cysteine, and ascorbate. Superoxide dismutase (SOD) was evaluated as a method described by Van-Rossum et al. (1997). The above-homogenized mixture was placed under 15 W fluorescent lamps for 10 min to initiate the reaction. The absorbance was taken at 560 mm. Catalase (CAT) was determined by Luck method, where 50 μl of above-homogenized supernatant was mixed with 50 mM phosphate buffer and 3 ml of 20 mM H_2_O_2_ (Luck, 1974). The absorption was measured at 240 nm. Nakano and Asada method calculated ascorbate peroxidase (APX) (Nakano and Asada, 1981). Morphological characters were measured as described previously.

### 2.6 Statistical analysis

Analysis of variance (ANOVA) was determined to figure out differences among the treatments using SPSS software (11.5 version). The significant differences were determined with the help of least significant difference (LSD) as a post hoc analysis (Nie et al., 1975).

## 3 Results

The microbial inoculation benefits carrot growth parameters and other bio-physiological activities compared to control in a polyhouse pot experiment (Tables 3 and 4). AMF and *P*. *fluorescence* colonized plants showed better results in terms of growth patterns and mineral nutrition over control plants. There were significant differences among the means of seven treatment groups as compared to control (Table 1). The maximum shoot length (34.39 ± 1.53) of treated *D*. *carota* was found in T6, *P*. *fluorescence* + *G*. *gigantean* (P_f_ + G_G_) inoculants which also corresponded with shoot weight (115.47 ± 1.63), (Table 3). Similarly, the root length (68.02 ± 1.88) and root weight (190.22 ± 1.81) were maximum in the same treatment, i.e., T6 (Table 3), followed by T7, consortium treatment (shoot length – 25.23 ± 1.27; shoot weight – 92.59 ± 1.09; root length – 55.44 ± 1.05; root weight – 116.04 ± 1.29). The maximum chlorophyll content (chlorophyll *a* 35.12 ± 1.17; chlorophyll *b* 17.81 ± 1.48; Total chlorophyll – 52.93 ± 2.25) and carotenoids (28.34 ± 1.61) were determined by the P_f_ + G_G_ treatment followed by the consortium (Total chlorophyll – 47.78 ± 2.92; carotenoid – 26.28 ± 1.29) treatment (Table 4). The total chlorophyll and carotenoid content were highest in T6 treatment because of the highest acidic (26.71 ± 2.06) and alkaline phosphatase activity (34.91 ± 1.16). Due to increased phosphatase activity root (3.12 ± 0.15) and shoot phosphorus content (1.17 ± 0.19) were also found best in the combination of *P*. *fluorescence* + *G*. *gigantean*. This agrees with the highest AM number (141 ± 3.50) and colonization (78.6 ± 5.40), as shown in Table 3. In the experiment, the highest content of carbohydrate (13.38 ± 0.81) was too found in T6, followed by consortium treatment (Table 3).

**Table 3:** Effect of Bioinoculants on Morphological and Food storage parameters of *Daucus carota* in the pot experiment.

**Treatments**	**Shoot length (cm)**	**Shoot weight (g)**	**Root length (cm)**	**Root weight (g)**	**Carbohydrate (mg/100 mg FW)**	**AM spore number**	**AM root colonization (%)**
Control	9.86 ± 1.11 ^g^‡	3.95 ± 0.84 ^h^	13.38 ± 1.27 ^h^	13.24 ± 1.53 ^g^	6.76 ± 0.96^f^	0 ± 00 ^h^	0 ± 00^f^
G_m_†	15.28 ± 0.61^f^	8.11 ± 0.82 ^g^	23.56 ± 1.02 ^g^	31.82 ± 2.61^f^	8.63 ± 0.78^e^	64 ± 6.60 ^g^	41.2 ± 5.70^e^
P_f_	20.37 ± 0.98^d^	18.14 ± 0.71^e^	34.46 ± 1.57^e^	64.78 ± 1.08^d^	10.74 ± 0.94 ^cd^	87 ± 5.10^e^	58.4 ± 3.70^c^
G_G_	18.16 ± 0.77^e^	11.15 ± 0.68^f^	27.54 ± 1.46^f^	48.14 ± 1.73^e^	9.68 ± 0.73^de^	78 ± 6.30^f^	50.2 ± 3.90^d^
G_m_ + P_f_	23.32 ± 0.81^c^	72.08 ± 0.96^d^	48.74 ± 1.18^c^	115.46 ± 2.21^b^	12.04 ± 0.81^b^	119 ± 6.80^c^	62.6 ± 7.50^c^
G_m_ + G_G_	21.52 ± 1.06^d^	79.91 ± 0.71^c^	40.78 ± 2.55^d^	82.88 ± 1.91^c^	11.52 ± 0.75^bc^	96 ± 6.50^d^	61.8 ± 3.10^c^
P_f_ + G_G_	34.39 ± 1.53^a^	115.47 ± 1.63^a^	68.02 ± 1.88^a^	190.22 ± 1.81^a^	13.38 ± 0.81^a^	141 ± 3.50^a^	78.6 ± 5.40^a^
G_m_ + P_f_ + G_G_	25.23 ± 1.27^b^	92.59 ± 1.09^b^	55.44 ± 1.05^b^	116.04 ± 1.29^b^	12.55 ± 0.78^ab^	127 ± 5.20^b^	69.8 ± 3.90^b^
LSD (*P* ≤ 0.05)	1.35	1.261	2.031	2.35	35.31	7.04	6.02
ANOVA (7, 32)	238.07	130.67	648.28	4.78	1.06	328.49	133.85

†G m – Glomus mosseae, P f – *Pseudomonas fluorescens*, G G – Gigaspora gigantea ± – Standard deviation; ‡ column brackets preceded by the same letter are not substantially different; p ≤ 0.05 – LSD (least significant difference test); FW – Fresh Weight.

**Table 4:** Effect of Bioinoculants on Biochemical and Physiological attributes of *Daucus carota* in pot experiment.

**Treatments**	**Chlorophyll *a* (mg FW-g)**	**Chlorophyll *b* (mg FW-g)**	**Total chlorophyll (mg FW-g)**	**Total carotenoids (mg FW-g)**	**Shoot phosphorus content %**	**Root phosphorus content %**	**Acid phosphatase (IU g-1 FW)**	**Alkaline phosphatase (IU g-1 FW)**
Control	11.52 ± 1.67 ^g^‡	5.29 ± 1.12 ^g^	16.81 ± 2.27 ^g^	10.21 ± 0.86 ^g^	0.27 ± 0.07^e^	0.71 ± 0.12 ^g^	6.68 ± 0.81 ^g^	10.17 ± 1.02 ^h^
G_m_†	18.51 ± 1.49^f^	8.61 ± 1.25^f^	27.12 ± 1.32^f^	15.67 ± 0.74^f^	0.55 ± 0.07^d^	0.98 ± 0.09^f^	9.79 ± 1.41^f^	15.25 ± 1.23 ^g^
P_f_	25.49 ± 1.74^d^	12.85 ± 1.05^d^	38.34 ± 2.03^d^	21.15 ± 0.79^d^	0.77 ± 0.17^bc^	1.88 ± 0.11^d^	13.42 ± 0.95^e^	19.94 ± 1.18^e^
G_G_	22.29 ± 2.08^e^	10.54 ± 1.84^e^	32.84 ± 3.21^e^	18.66 ± 0.81^e^	0.67 ± 0.06 ^cd^	1.22 ± 0.15^e^	11.91 ± 1.14^e^	17.18 ± 0.84^f^
G_m_ + P_f_	28.43 ± 1.31^c^	13.23 ± 1.25 ^cd^	41.67 ± 2.44^c^	24.67 ± 0.72^c^	0.83 ± 0.07^bc^	2.44 ± 0.17^bc^	17.54 ± 0.83^c^	23.87 ± 1.21^c^
G_m_ + G_G_	26.14 ± 1.82^d^	14.86 ± 1.06^bc^	41.01 ± 2.48 ^cd^	24.56 ± 0.91^c^	0.79 ± 0.10^bc^	2.35 ± 0.33^c^	15.66 ± 0.93^d^	21.99 ± 1.25^d^
P_f_ + G_G_	35.12 ± 1.17^a^	17.81 ± 1.48^a^	52.93 ± 2.25^a^	28.34 ± 1.61^a^	1.17 ± 0.19^a^	3.12 ± 0.15^a^	26.71 ± 2.06^a^	34.91 ± 1.16^a^
G_m_ + P_f_ + G_G_	32.43 ± 1.39^b^	15.34 ± 1.96^b^	47.78 ± 2.92^b^	26.28 ± 1.29^b^	0.94 ± 0.16^b^	2.64 ± 0.14^b^	19.73 ± 1.64^b^	25.43 ± 0.86^b^
LSD (*P* ≤ 0.05)	2.07	1.83	3.12	1.31	0.16	0.21	1.66	1.42
ANOVA (7, 32)	110.56	40.06	112.78	180.08	22.86	131.64	116.67	225.26

†G m – Glomus mosseae, P f – *Pseudomonas fluorescens*, G G – Gigaspora gigantea ± – Standard deviation; ‡ column brackets preceded by the same letter are not substantially different; p ≤ 0.05 – LSD (least significant difference test); FW – Fresh Weight

**Fig. 1:**
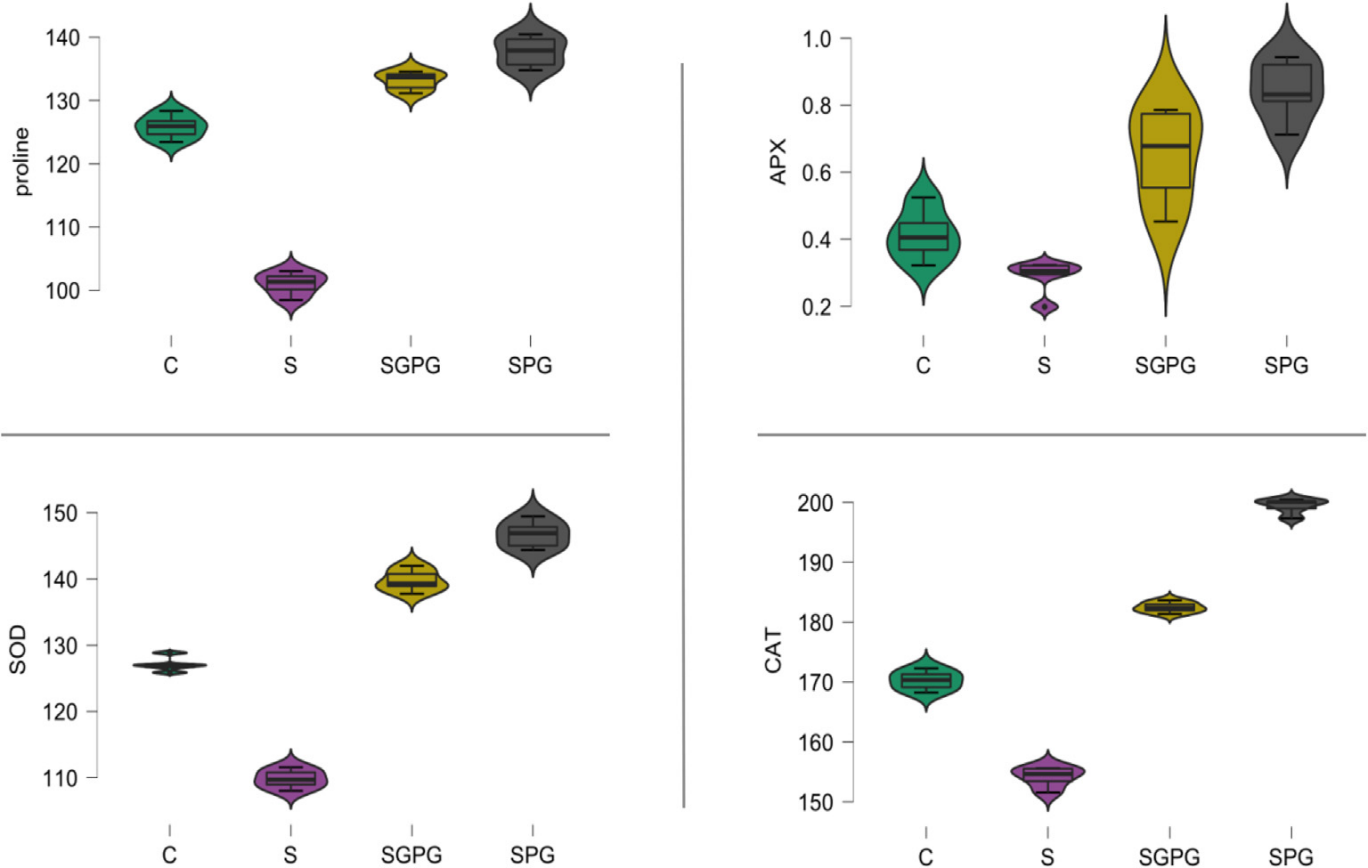
Variation for the proline (μmol g^−1^ FW), Superoxide dismutase (SOD; U mg^−1^ protein), Ascorbate peroxidase (APX; mg protein min-10) and Catalase (CAT; U mg^−1^ protein) in the salt screening experiment, where C: Control; S: Salt stress with no inoculum; SGPG: Salt stress with *Glomus mosseae*, *Pseudomonas fluorescens*, *Gigaspora gigantea*; SPG: Salt stress with *Glomus mosseae* and *Pseudomonas fluorescens*. Salt stress – 150 mM NaCl (after 10 days of germination).

In the case of field experiments, the best two treatments from the previous pot experiment were selected. This experiment deals with salt stress given to the plants. The plants with no salt and no microbial inoculum, i.e., T1, showed better results than the plants with only salt stress T2, as shown in Table 5, Table 6, Table 7. The plants with *Pseudomonas fluorescens* and *Gigaspora gigantea,* i.e., T3 showed better results than with consortium treatment (T4) in the field experiment. The shoot length (15.39 ± 1.66) and weight (13.27 ± 1.89) were highest in T3 as compared to T4 (shoot length – 13.27 ± 1.89; shoot weight – 12.78 ± 1.38) which also corresponds to maximum Root length (26.03 ± 1.91) and weight (27.03 ± 1.91) in T3 (Table 5). Total chlorophyll (chlorophyll *a* – 37.13 ± 2.31; chlorophyll *b* – 18.42 ± 1.96; Total chlorophyll – 48.78 ± 2.88) and carotenoid (31.82 ± 1.17) in T3 were also found a maximum that linked with the carbohydrate content. The carbohydrate content (15.38 ± 1.523) in carrot was highest in T3 because the phosphatase enzymes (acid phosphatase – 25.32 ± 1.71; alkaline phosphatase – 34.91 ± 1.16) that increase the phosphorus content was superlative in T3 (Table 6). Similarly the content of root (3.26 ± 0.18) and shoot phosphorus (1.35 ± 0.13) were also best in T3 (Table 6). This is all because of the highest colonization (68.40 ± 4.03) and higher number of AMF (117.40 ± 6.10) in this treatment, i.e., the combination of P_f_ + G_G_. Whereas various stress-relieving enzymes were found highest in P_f_ + G_G_; T3 (Fig. 1). The three most essential enzymes, namely catalase (182.47 ± 0.88), ascorbate peroxidase (0.64 ± 0.14) and superoxide dismutase (139.73 ± 1.64), were found to be highest in T3 where salt stress is given along with P_f_ + G_G_. In the case of T3, the electrolyte leakage (32.71 ± 1.96) and peroxides, a reactive oxygen species (15.25 ± 1.23) was least signifying that of Pf + G_G_ did not allow them to produce in more concentration due to high water intake and accelerated CAT, ASP and SOD (Table 7; Fig. 1). In such cases, the proline content (137.69 ± 2.45), which is found more during stress conditions as it decreases the osmotic potential and increases the water intake, was also found to be the highest in T3. Surprisingly, the consortium treatment was not reported as efficient (Peroxide – 10.17 ± 1.02; Electrolyte leakage – 35.74 ± 1.19; Proline – 133.18 ± 1.46; CAT-182.47 ± 0.88; APX – 0.64 ± 0.14; SOD – 139.31.64) (Fig. 1).

**Table 5:** Effect of Bioinoculants on Morphological and Food storage parameters of *Daucus carota* in open field condition.

**Treatments**	**Shoot length (cm)**	**Shoot weight (g)**	**Root weight (g)**	**Carbohydrate (mg/100 mg FW)**	**AM spore number**	**AM root colonization (%)**
Control	9.66 ± 1.04^c^‡	9.02 ± 1.82^c^	14.87 ± 1.12^b^	11.75 ± 1.02^c^	20.40 ± 2.07^c^	16.80 ± 3.56^b^
Salt^#^	8.28 ± 0.94^d^	7.08 ± 1.56^d^	11.92 ± 1.32^c^	7.93 ± 1.08^d^	19.20 ± 2.38^d^	11.40 ± 2.32^c^
Salt + P_f_ + G_G_†	15.39 ± 1.66^a^	15.47 ± 1.642^a^	27.03 ± 1.910^a^	15.38 ± 1.523^a^	117.40 ± 6.10^a^	68.40 ± 4.03^a^
Salt + G_m_ + P_f_ + G_G_	13.27 ± 1.89^b^	12.78 ± 1.38^b^	26.04 ± 1.74^a^	13.55 ± 1.34^b^	103.20 ± 7.05^b^	65.80 ± 4.32^a^
LSD (*P* ≤ 0.05)	1.93	2.16	2.08	1.66	4.88	6.61
ANOVA (3, 16)	25.52	27.17	121.74	32.56	355.59	566.96

†G m – *Glomus mosseae*, P f – *Pseudomonas fluorescens*, G G – *Gigaspora gigantea* ± – Standard deviation; ‡ column brackets preceded by the same letter are not substantially different; p ≤ 0.05 – LSD (least significant difference test); FW – Fresh Weight.

**Table 6:** Effect of Bioinoculants on Biochemical and Physiological attributes of *Daucus carota* in open field condition.

**Treatments**	**Chlorophyll *a* (mg FW^-g^)**	**Chlorophyll *b* (mg FW^-g^)**	**Total chlorophyll**	**Total carotenoids**	**Shoot phosphorus content**	**Root phosphorus content**	**Acid Phosphatase**	**Alkaline Phosphatase**
			**(mg FW^-g^)**	**(mg FW^-g^)**	**%**	**%**	**(IU g^−1^ FW)**	**(IU g^−1^ FW)**
**Control**	**21.52 ± 1.67^c^‡**	11.69 ± 1.65^c^	33.21 ± 1.08^c^	16.46 ± 1.95^b^	0.61 ± 0.08^c^	0.99 ± 0.198^c^	11.29 ± 0.669^c^	10.17 ± 1.02^c^
Salt^#^	17.51 ± 1.41^d^	8.41 ± 1.01^d^	25.92 ± 1.44^d^	12.87 ± 1.65^c^	0.51 ± 0.06^d^	0.84 ± 0.09	10.47 ± 0.987^d^	15.25 ± 1.23^d^
Salt + P_f_ + G_G_†	37.13 ± 2.31^a^	18.42 ± 1.96^a^	55.54 ± 3.74^a^	31.82 ± 1.17^a^	1.35 ± 0.13^a^	3.26 ± 0.18^a^	25.32 ± 1.71^a^	34.91 ± 1.16^a^
Salt + G_m_ + P_f_ + G_G_	33.83 ± 2.17^b^	14.94 ± 1.51^b^	48.78 ± 2.88^b^	29.88 ± 0.85^a^	1.06 ± 0.18^b^	2.74 ± 0.26^b^	19.69 ± 1.67^b^	25.43 ± 0.86^b^
LSD (*P* ≤ 0.05)	2.58	2.11	2.25	1.97	0.17	0.26	1.79	1.45
ANOVA (3, 16)	120.05	37.12	145.95	208.59	48.01	195.44	141.19	513.14

†G m – *Glomus mosseae*, P f – *Pseudomonas fluorescens*, G G – Gigaspora gigantea ± – Standard deviation; ‡ column brackets preceded by the same letter are not substantially different; p ≤ 0.05 – LSD (least significant difference test); FW – Fresh Weight

**Table 7:** Effect of Bioinoculants on Stress Physiological attributes of *Daucus carota* in open field condition.

**Treatments**	**Peroxide content (μmol g-1 FW)**	**Electrolyte leakage (%)**	**Catalase (U mg^−1^ protein)**
Control	25.43 ± 0.86^c^‡	40.17 ± 1.02^c^	170.27 ± 1.621^c^
Salt^#^	34.91 ± 1.16^d^	45.05 ± 1.16^d^	154.15 ± 1.69^d^
Salt + P_f_ + G_G_†	15.25 ± 1.23^a^	32.71 ± 1.96^a^	199.37 ± 1.27^a^
Salt + G_m_ + P_f_ + G_G_	10.17 ± 1.02^b^	35.74 ± 1.19^b^	182.47 ± 0.88^b^
LSD (*P* ≤ 0.05)	1.45	1.86	1.88
ANOVA (3, 16)	513.14	75.05	925.62

†G m – *Glomus mosseae*, P f – *Pseudomonas fluorescens*, G G – *Gigaspora gigantea* ± – Standard deviation; ‡ column brackets preceded by the same letter are not substantially different; p ≤ 0.05 – LSD (least significant difference test); FW – Fresh Weight.

## 4 Discussion

The present work describes the capacity of beneficial microbes in the vegetable production systems. A significant effect on carrot cultivated under salinity stress in field and greenhouse conditions utilizing AMF and *Pseudomonas* has proven their effectiveness (Evelin et al., 2019). Soil microbes regularly improve soil biodiversity by changing the unfavorable environment. AMF are obligate symbionts, where fungal associates assist the host by improving the uptake of water and minerals (Saini et al., 2019a). Preceding work on other root crops confirmed the progressive impact of AMF on growth and yield (Yarzábal and Chica, 2017, Ahmad and Gucel, 2016). AMF and bacteria can be beneficial for each other and help plants access mineral nutrients, especially phosphorus (Saini et al., 2019b, Saini et al., 2020a, Saini et al., 2020b). It is useful to recommend that AMF in the rhizosphere can have an additive effect on carrot development. The plant growth parameters increased in our results; this might be due to enhancing phytohormones, especially auxin, gibberellins, and cytokines (Duca et al., 2014, Ahmad et al., 2015a, Ahmad et al., 2015b). Besides this, there is the formation of an Iron-chelating substance, “siderophores” which are further useful in increasing photosynthesis and respiration in plants (Jilling et al., 2018).

AMF and PSB inoculated plants showed better growth and more acquisition of nutrients and water, making the plants healthy during salt stress compared to the control plants and plants having only salt concentration. AMF is also known to decrease the cellular dehydration by upholding the turgor pressure (Zou et al., 2013). AMF pre-inoculated seeds showed better growth in terms of plant weight. It might be due to higher photosynthetic efficiency by increasing chlorophyll content and lipids (Berruti et al., 2018). It is well known photosynthetic pigments disrupt due to salinity. Still, in present investigation, AMF mediated seeds overcome this destruction by absorbing more Mg and N via AM hyphae mycorrhizal that can penetrate at deeper lengths (Begum et al., 2019). Higher biomass can also lead to dilution of sodium and chlorine and further prove to be better for the crop (Talaat and Shawky, 2014). Other plant growth was significantly better under AMF and PSB conditions; this may be due to higher procurement of water and nutrients status due to great root architecture (Talaat and Shawky, 2014, Chang et al., 2018).

On the same lines, the osmotic balance of the carrot is maintained by AMF further accumulated osmo-regulators and antioxidants which ought to be the reason why AMF colonized seeds ascertain better growth even under moderate level of salinity (Chandrasekaran et al., 2014). Moreover, the scavenging system is highly activated in AMF pre-colonized plants, which helps eliminate reactive oxygen species (ROS) and, hence, averts the oxidative stress. The result in present study showed the increase in antioxidant enzymes which is also confirmed by Begum et al. (2019) that during AMF inoculation, there is increased synthesis of SOD, CAT and POX. Similarly, Mishra et al., (2018) concluded that AMF colonized vegetable crops have a high number of antioxidants like carotenoids, GSH, α-tocopherols, and AsA in plants that can directly scavenge ·O_2_^–^, H_2_O_2_ and another ROS (Evelin and Kapoor, 2014). AMF modulates salt stress by increasing K^+^ accretion, facilitating the crop to sustain a lower Na^+^/K^+^ ratio that helps in circumventing mutilation to the biological functioning (Chen et al., 2017). Hashem et al. (2014) also confirm an increase in antioxidant enzyme activities with an increase in salinity stress, stating that AMF mediated plants could have a higher membrane stability. The decrease in the electrolyte leakage was shown in AMF mediated plants that may be attributed to enhancing nutrient uptake, osmotic homeostasis, and dilution of ions' toxic effects (Campanelli et al., 2013, Zhao et al., 2010).

The microbial inoculation extends aquaporins that promote the diffusion of CO_2_ in mesophyll cells, which further protect the photosynthetic apparatus (Chen et al., 2017). This is correlated with enhanced RuBisCO activity in AMF inoculated plants which is due to increased RuBisCo (Chen et al., 2017). AMF can also facilitate crop plants to retract sodium ions from xylem tissues and sidetrack it away from the mesophyll cells to roots (Maathuis, 2013). Moreover, AMF inoculated plants can produce an advantage molecule like glomalin, a glycoprotein which can be regarded as heat shock protein (HSP_60_) (Hammer and Rillig, 2011). It is also believed that glomalin can participate in tolerating cytosolic damages during abiotic stresses; this also adds in conforming out results (Begum et al., 2019, Hammer and Rillig, 2011).

AMF inoculated plants can also alleviate salt stress by compartmentalizing sodium ion into the vacuole by regulating OsNHX_3_ (sodium/hydrogen exchanger) (Porcel et al., 2016). In present study, there is an increase in proline content during salt stress, but the AMF plants showed a higher concentration of proline than the control (Abo-Doma et al., 2016). Ait-El-Mokhtar et al. (2020) also confirms that after the inoculation of AMF under salt stress proline content increases. There is upregulation of P_5_CS gene during AMF inoculation as well as glutamate dehydrogenase which synthesize glutamate, the precursor of proline. Hence there is more expression of proline as well as there is inactivation of proline dehydrogenase (Malhi et al., 2021, Jan et al., 2018).

AMF ameliorates the phytohormones production so gibberellins, salicylic acid (SA) impart a positive response for the plant during salt stress, where a foliar spray of GA promotes salinity tolerance (Garg *et al.,* 2018). SA also induces ionic balance by modulating carbohydrate metabolism, reducing lipid peroxidation, and increasing reduced sugars, proline, proteins K^+^ ions, etc. (Garg and Bharti, 2018). In present study, amount of carotenoids also showed a significant increase which is also proved to be antioxidant as they prevent ·O_2_^–^ production (Ramel et al., 2012). Increased SOD might also be why microbial inoculated plants showed less stress, or we can say combat the salt stress by dissolving peroxides (Giannopolitis and Ries, 1977, Kaur et al., 2018). Ebrahim *et al.* (2017) also confirm that inoculating microbial strain in tomato field salt stress is relieved, especially by AMF (Ebrahim and Saleem 2017). In other crops like medicinal plants (*Medicago truncatula*), *Rhizophagus irregularis* an AMF solubilize K^+^ sources under K^+^ deficiency (Garcia et al., 2017).

## 5 Conclusion

AMF treatment and the Pseudomonas appear to be promising for the carrot in nutrient procurement and maintaining ionic homeostasis. In this direction, the AMF seems to be in a appropriate inoculum to improve plant development, yield, and quality especially in the case of carrots. Although, an optimum values for the biochemical and the morphological traits have been based on the treatment with *P. fluorescens*, and *G. gigantea*. Overall, this work highlights microbial inoculum and mycorrhizal fungi possess an efficacy in attaining a profitable carrot production by improving water uptake, maintaining osmotic balance, enhancing photosynthetic efficiency, and modulating phytohormones profiling. Results of this study also established that AMF and PSB application enhance the quality of carrots.

## Declaration of Competing Interest

The authors declare that they have no known competing financial interests or personal relationships that could have appeared to influence the work reported in this paper.

## References

[b0005] Abo-Doma A., Edrees S., Abdel-Aziz S.H. (2016). The effect of mycorrhiza growth and expression of some genes in barley. Egypt. J. Genet. Cytol..

[b0010] Ahmad P., Hashem A., Abde-Allah E.F., Alqarawi A.A., John R. (2015). Egamberdieva, D. Role of *Trichoderma harzianum* in mitigating NaCl stress in Indian mustard (*Brassica juncea* L.) through antioxidative defense system. Front. Plant Sci..

[b0015] Ahmad P., Jaleel C.A., Salem M.A., Nabi G., Sharma S. (2010). Roles of enzymatic and nonenzymatic antioxidants in plants during abiotic stress. Crit. Rev. Biotechnol..

[b0020] Ahmad P., Sarwat M., Bhat N.A., Wani M.R., Kazi A.G., Tran L.-S.P. (2015). Alleviation of cadmium toxicity in Brassica juncea L. (Czern. & Coss.) by calcium application involves various physiological and biochemical strategies. PLoS ONE.

[b0025] Ahmad P., Gucel S. (2016). Mitigation of NaCl stress by arbuscular mycorrhizal fungi through the modulation of osmolytes, antioxidants and secondary metabolites in mustard (Brassica juncea L.) plants. Front. Plant Sci..

[b0030] Ahmad P., Ahanger M.A., Alyemeni M.N., Wijaya L., Egamberdieva D., Bhardwaj R., Ashraf M. (2017). Zinc application mitigates the adverse effects of NaCl stress on mustard [Brassica juncea (L.) Czern & Coss] through modulating compatible organic solutes, antioxidant enzymes, and flavonoid content. J. Plant Interact..

[b0035] Ahmad P., Ahanger M.A., Alam P., Alyemeni M.N., Wijaya L., Ali S., Ashraf M. (2019). Silicon (Si) supplementation alleviates NaCl toxicity in mung bean [Vigna radiata (L.) Wilczek] through the modifications of physio-biochemical attributes and key antioxidant enzymes. J. Plant Growth Regul..

[b0040] Ahanger M.A., Bhat J.A., Siddiqui M.H., Rinklebe J., Ahmad P. (2020). Integration of silicon and secondary metabolites in plants: a significant association in stress tolerance. J. Exp. Bot..

[b0045] Ait-El-Mokhtar M., Baslam M., Ben-Laouane R., Anli M., Boutasknit A., Mitsui T., Wahbi S., Meddich A. (2020). Salt Stress on date palm (*Phoenix dactylifera* L.) by the application of arbuscular mycorrhizal fungi and/or compost. Front. Sustain. Food Syst..

[b0050] Arnon D.I. (1949). Copper enzymes in isolated chloroplasts polyphenol oxidase in *Beta vulgaris*. Plant Physiol..

[b0055] Bandyopadhyay K., Aggarwal P., Chakraborty D., Pradhan S., Narayan Garg R., Singh R. (2012). Practical Manual on Measurement of Soil Physical Properties Practical.

[b0060] Bano S., Ashraf M., Akram N.A., Al-Qurainy F. (2012). Regulation in some vital physiological attributes and antioxidative defense system in carrot (*Daucus carota* L.) under saline stress. J. Appl. Bot. Food Qual..

[b0065] Bates I.S., Waldren R.P., Teare I.D. (1973). Rapid determination of free proline for water stress studies. Plant Soil.

[b0070] Begum N., Ahanger M.A., Su Y., Lei Y., Mustafa N.S.A., Ahmad P., Zhang L. (2019). Improved drought tolerance by AMF inoculation in maize (Zea mays) involves physiological and biochemical implications. Plants.

[b0075] Berruti A., Bianciotto V., Lumini E. (2018). Seasonal variation in winter wheat field soil arbuscular mycorrhizal fungus communities after non-mycorrhizal crop cultivation. Mycorrhiza.

[b0080] Campanelli A., Ruta C., DeMastro G., Morone-Fortunato I. (2013). The role of arbuscular mycorrhizal fungi in alleviating salt stress in Medicago sativa L. Car. Icon. Symbiosis..

[b0085] Chandrasekaran M., Boughattas S., Hu S.J., Oh S., Sa T.M. (2014). A meta-analysis of arbuscular mycorrhizal effects on plants grown under salt stress. Mycorrhiza.

[b0090] Chang W., Sui X., Fan X., Jia T.T., Song F.Q. (2018). Arbuscular mycorrhizal symbiosis modulates antioxidant response and ion distribution in salt-stressed *Elaeagnus angustifolia* seedlings. *Front. Microbiol*..

[b0095] Chen J., Zhang H., Zhang X., Tang M. (2017). Arbuscular mycorrhizal symbiosis alleviates salt stress in black locust through improved photosynthesis, water status, and K^+^/Na^+^ homeostasis. Front. Plant Sci..

[b0100] Dionisio-Sese M.L., Tobita S. (1998). Antioxidant responses of rice seedlings to salinity stress. Plant Sci..

[b0105] Duca D., Lorv J., Patten C.L., Rose D., Glick B.R. (2014). Indole-3-acetic acid in plant-microbe interactions. Anton Van Leeuw..

[b0110] Ebrahim M.K.H., Saleem A. (2017). Alleviating salt stress in tomato inoculated with mycorrhizae: photosynthetic performance and enzymatic antioxidants. J. Taibah Univ. Sci..

[b0115] Evelin H., Devi T.S., Gupta S., Kapoor R. (2019). Mitigation of salinity stress in plants by arbuscular mycorrhizal symbiosis: current understanding and new challenges. Front. Plant Sci..

[b0120] Evelin H., Kapoor R. (2014). Arbuscular mycorrhizal symbiosis modulates antioxidant response in salt-stressed *Trigonella foenum-graecum* Plants. Mycorrhiza.

[b0125] Füzy A., Kovács R., Cseresnyés I., Parádi I., Szili-Kovács T., Kelemen B., Rajkai K., Takács T. (2019). Selection of plant physiological parameters to detect stress effects in pot experiments using principal component analysis. Acta Physiol. Plant..

[b0130] Garcia K., Chasman D., Roy S., Ane J.M. (2017). Physiological responses and gene co-expression network of mycorrhizal roots under K^+^ deprivation. Plant Physiol..

[b0135] Garg N., Bharti A. (2018). Salicylic acid improves arbuscular mycorrhizal symbiosis, and chickpea growth and yield by modulating carbohydrate metabolism under salt stress. Mycorrhiza.

[b0140] Giannopolitis C.N., Ries S.K. (1977). Superoxide dismutases I. Occurrence in higher plants. *Plant Physiol*..

[b0145] Giovannetti M., Mosse B. (1980). An evaluation of techniques for measuring vesicular arbuscular mycorrhizal infection in roots. New Phytol..

[b0150] Hammer E.C., Rillig M.C. (2011). The influence of different stresses on glomalin levels in an arbuscular mycorrhizal fungus-salinity increases glomalin content. PLoS ONE.

[b0155] Hashem A., Abde-Allah E.F., Alqarawi A.A., El-Didamony G., Alwhibi Mona S., Egamberdieva D., Ahmad P. (2014). Alleviation of ad-verse impact of salinity on faba bean (Vicia faba L.) by arbuscular mycorrhizal fungi. Pak. J. Bot..

[b0160] Jacoby R., Peukert M., Succurro A., Koprivova A., Kopriva S. (2017). The role of soil microorganisms in plant mineral nutrition-current knowledge and future directions. Front. Plant Sci..

[b0165] Jan S., Alyemeni M.N., Wijaya L., Alam P., Siddique K.H., Ahmad P. (2018). Interactive effect of 24-epibrassinolide and silicon alleviates cadmium stress via the modulation of antioxidant defense and glyoxalase systems and macronutrient content in Pisum sativum L. seedlings. BMC Plant Biol..

[b0170] Jilling A., Keiluweit M., Contosta A.R., Frey S., Schimel J., Schnecker J., Smith R.G., Tiemann L., Stuart-Grandy A. (2018). Minerals in the rhizosphere: Overlooked mediators of soil nitrogen availability to plants and microbes. Biogeochemistry.

[b0175] Kaur H., Sirhindi G., Bhardwaj R., Alyemeni M.N., Siddique K.H., Ahmad P. (2018). 28-homobrassinolide regulates antioxidant enzyme activities and gene expression in response to salt-and temperature-induced oxidative stress in Brassica juncea. Sci. Rep..

[b0180] Kaushik P., Sandhu O.S., Brar N.S., Kumar V., Malhi G.S., Kesh Hari, Saini I. (2020). Soil metagenomics: prospects and challenges. in: mycorrhizal fungi-utilization in agriculture and industry. *IntechOpen*.

[b0185] Kiers E.T., West S.A. (2015). Evolving new organisms via symbiosis. Science.

[b0190] Liu S., Guo X., Feng G., Maimaitiaili B., Fan J., He X. (2016). Indigenous arbuscular mycorrhizal fungi can alleviate salt stress and promote growth of cotton and maize in saline fields. Plant Soil.

[b0195] Luck H. (1974). Estimation of catalase, methods in enzymatic analysis.

[b0200] Maathuis F.J. (2013). Sodium in plants: Perception, signalling, and regulation of sodium fluxes. J. Exp. Bot..

[b0205] Malhi G.S., Kaur M., Kaushik P., Alyemeni M.N., Alsahli A.A., Ahmad P. (2021). Arbuscular mycorrhiza in combating abiotic stresses in vegetables: An eco-friendly approach. Saudi J. Biol. Sci..

[b0210] Massa N., Cesaro P., Todeschini V., Capraro J., Scarafoni A., Cantamessa S., Copetta A., Anastasia F., Gamalero E., Lingua G. (2020). Selected autochthonous rhizobia, applied in combination with AM fungi, improve seed quality of common bean cultivated in reduced fertilization condition. Appl. Soil Ecol..

[b0215] Mishra V., Ellouze W., Howard R.J. (2018). Utility of arbuscular mycorrhizal fungi for improved production and disease mitigation in organic and hydroponic greenhouse crops. J. Hortic..

[b0220] Nakano Y., Asada K. (1981). Hydrogen peroxide is scavenged by ascorbate-specific peroxidase in spinach chloroplasts. Plant Cell Physiol..

[b0225] Navarro J.M., Perez-Tornero O., Morte A. (2013). Alleviation of salt stress in citrus seedlings inoculated with arbuscular mycorrhizal fungi depends on the rootstock salt tolerance. J. Plant Physiol..

[b0230] Nie N.H., Bent D.H., Hull C.H. (1975). SPSS: Statistical Package for the Social Sciences.

[b0235] Phillips J.M., Hayman D.S. (1970). Improved procedures for clearing roots and staining parasitic and vesicular-arbuscular mycorrhizal fungi for rapid assessment of infection. Trans Brit. Mycol. Soc..

[b0240] Porcel R., Aroca R., Azcon R., Ruiz-Lozano J.M. (2016). Regulation of cation transporter genes by the arbuscular mycorrhizal symbiosis in rice plants subjected to salinity suggests improved salt tolerance due to reduced Na^+^ root-to-shoot distribution. Mycorrhiza.

[b0245] Pozo M.J., Lopez-Raez J.A., Azcon-Aguilar C., Garcia-Garrido J.M. (2015). Phytohormones as integrators of environmental signals in the regulation of mycorrhizal symbioses. New Phytol..

[b0250] Ramel F., Birtic S., Ginies C., Soubigou-Taconnat L., Triantaphylidès C., Havaux M. (2012). Carotenoid oxidation products are stress signals that mediate gene responses to singlet oxygen in plants. *Proc. Natl. Acad. Sci.* U.S.A..

[b0255] Regvar M., Vogel-Mikus K., Ševerkar T. (2003). Effect of AMF inoculum from field isolates on the yield of green pepper, parsley, carrot, and tomato. Folia Geobot..

[b0260] Saini I., Aggarwal A., Kaushik P. (2019). Influence of biostimulants on important traits of *Zinnia elegans* jacq. under open field conditions. Int. J. Agron..

[b0265] Saini I., Aggarwal A., Kaushik P. (2019). Inoculation with mycorrhizal fungi and other microbes to improve the morpho-physiological and floral traits of *Gazania rigens* (L.) Gaertn. Agriculture.

[b0270] Saini I., Himanshi Rani K., Gill N., Sandhu K., Bisht N., Kumar T., Kaushik P. (2020). Significance of arbuscular mycorrhizal fungi for Acacia: A Review. Pak. J. Biol. Sci..

[b0275] Saini I., Yadav V.K., Kaushik P. (2020). Effect of superphosphate, urea and bioinoculants on *Zinnia elegans* Jacq. Ind. J. Exp. Biol..

[b0280] Sbrana C., Avio L., Giovannetti M. (2014). Beneficial mycorrhizal symbionts affecting the production of health-promoting phytochemicals. Electrophoresis.

[b0285] Sharma M., Kaushik P., Chaturvedi P. (2018). Enumeration, antagonism, and enzymatic activities of microorganisms isolated from railway station soil. BioRxiv.

[b0290] Shrivastava P., Kumar R. (2015). Soil salinity: A serious environmental issue and plant growth promoting bacteria as one of the tools for its alleviation. Saudi J. Biol. Sci..

[b0295] Simon P., Iorizzo M., Grzebelus D., Baranski R. (2019). The Carrot Genome.

[b0305] Stolarczyk J., Janick J. (2011). Carrot: History and iconography. Chron..

[b0310] Talaat N.B., Shawky B.T. (2014). Protective effects of arbuscular mycorrhizal fungi on wheat (*Triticuma estivum* L.) plants exposed to salinity. Environ. Exp. Bot..

[b0315] Van-Rossum M.W.P.C., Alberda M., Van der-Plas L.H.W. (1997). Role of oxidative damage in tulip bulb scale micropropagation. Plant Sci..

[b0320] Velikova V., Yordanov I., Edreva A. (2000). Oxidative stress and some antioxidant system in acid rain treated bean plants: protective role of exogenous polyamines. Plant Sci..

[b0325] Verbon E.H., Liberman L.M. (2016). Beneficial microbes affect endogenous mechanisms controlling root development. Trends Plant Sci..

[b0330] Wang Y., Wang M., Li Y., Wu A., Huang J. (2018). Effects of arbuscular mycorrhizal fungi on growth and nitrogen uptake of *Chrysanthemum morifolium* under salt stress. PLoS One.

[b0335] Xia Y., Debolt S., Dreyer J., Scott D., Williams M.A. (2015). Characterization of culturable bacterial endophytes and their capacity to promote plant growth from plants grown using organic or conventional practices. Front. Plant Sci..

[b0340] Yarzábal L.A., Chica E.J. (2017). Potential for developing low-input sustainable agriculture in the tropical Andes by making use of native microbial resources. Plant-Microbe Interactions in Agro-Ecological Perspectives.

[b0345] Zhao M., Li M., Liu R.J. (2010). Effects of arbuscular mycorrhizae on microbial population and enzyme activity in replant soil used for watermelon production. Int. J. Eng. Sci. Tech..

[b0350] Zou Y.N., Wu Q.S., Huang Y.M., Ni Q.D., He X.H. (2013). Mycorrhizal-mediated lower proline accumulation in Poncirus trifoliata under water deficit derives from the integration of inhibition of proline synthesis with increase of proline degradation. PLoS ONE.

